# Initial Maximum Overlap Method Embedded with Extremely Localized Molecular Orbitals for Core-Ionized States of Large Systems

**DOI:** 10.3390/molecules28010136

**Published:** 2022-12-24

**Authors:** Giovanni Macetti, Alessandro Genoni

**Affiliations:** 1 Université de Lorraine & CNRS, Laboratoire de Physique et Chimie Théoriques (LPCT), UMR CNRS 7019, 1 Boulevard Arago, 57078 Metz, France; 2 Dipartimento di Chimica, Università degli Studi di Milano, Via Golgi 19, 20133 Milano, Italy

**Keywords:** core-ionized states, embedding techniques, extremely localized molecular orbitals (ELMOs), initial maximum overlap method (IMOM), ΔSCF techniques, large (bio)systems

## Abstract

Despite great advances in X-ray absorption spectroscopy for the investigation of small molecule electronic structure, the application to biosystems of experimental techniques developed within this research field remains a challenge. To partially circumvent the problem, users resort to theoretical methods to interpret or predict the X-ray absorption spectra of large molecules. To accomplish this task, only low-cost computational strategies can be exploited. For this reason, some of them are single Slater determinant wavefunction approaches coupled with multiscale embedding techniques designed to treat large systems of biological interest. Therefore, in this work, we propose to apply the recently developed IMOM/ELMO embedding method to the determination of core-ionized states. The IMOM/ELMO technique resulted from the combination of the single Slater determinant Δself-consistent-field-initial maximum overlap approach (ΔSCF-IMOM) with the QM/ELMO (quantum mechanics/extremely localized molecular orbital) embedding strategy, a method where only the chemically relevant region of the examined system is treated at fully quantum chemical level, while the rest is described through transferred and frozen extremely localized molecular orbitals (ELMOs). The IMOM/ELMO technique was initially validated by computing core-ionization energies for small molecules, and it was afterwards exploited to study larger biosystems. The obtained results are in line with those reported in previous studies that applied alternative ΔSCF approaches. This makes us envisage a possible future application of the proposed method to the interpretation of X-ray absorption spectra of large molecules.

## 1. Introduction

X-ray absorption spectroscopy is a very useful experimental method to obtain information on the nature of molecules and on the features of their chemical bonds. In this context, examples of very well-known techniques are the near-edge X-ray absorption fine structure spectra (NEXAFS) and the inner shell electron energy loss spectroscopy (EELS). However, if on the one hand the interpretation of the X-ray absorption spectra is relatively simple when small systems are investigated [1,2,3,4,5,6,7], on the other hand it becomes more and more intricated when large biological molecules (e.g., proteins) are considered. The latter cases are also further complicated by effects due to the chemical environment, which usually plays a non-negligible role.

To overcome the drawback, different experimental strategies have been proposed over the years. The simplest one consists in computing and analyzing spectral differences between proteins. Another technique is the building block approach (BBA) [8,9], which is based on the experimental observation that di- or tripeptide spectra are given by the sum of the individual spectra associated with the single constituting amino acids [10,11]. Nevertheless, although somehow useful, the BBA method unfortunately neglects aspects that were indicated by computational studies as crucial to understand the X-ray absorption spectra of biological molecules: protonation states of residues, conformational effects (e.g., secondary structure of proteins), and non-covalent interactions [12,13,14,15]. For this reason, nowadays the use of fully theoretical methods is probably the most advantageous approach to try to interpret or predict the X-ray absorption spectra of macromolecular systems of biological interest, although, to achieve the same goal, one could also imagine exploiting the routines contained in very well-tuned and advanced software packages (e.g., *SHELX* [16]) that have assisted crystallographers in their daily tasks for many years.

Anyway, to this purpose, today we have a plethora of theoretical methods that would allow the description of core-ionized states. Among them, the many-determinant wavefunction approaches are clearly the most accurate ones [17,18,19,20,21]. However, they are also the most time consuming. Therefore, they are not easily applicable to large molecules. On the contrary, the techniques based on a single Slater-determinant wavefunction *ansatz* are slightly less accurate, but much more computationally advantageous. They are consequently more suitable to treat biomolecules. For this reason, some of them will be briefly reviewed in the following paragraphs.

The simplest method is certainly the one relying on Koopman’s theorem [22], according to which the ionization energy ($E_{ion}$) is simply given by the negative of the orbital energy associated with the molecular orbital of interest. Nevertheless, if this technique provides results in very good agreement with experimental data for valence $E_{ion}$ values, it unfortunately leads to overestimated core ionization energies due to the lack of orbital relaxation and correlation treatment.

Another simple single-determinant technique is the so-called “Z + 1 scheme”, which is generally exploited to determine relative energies rather than absolute $E_{ion}$ values. This approach relies on the observation that we generally have an analogy between energy levels/valence properties of a core ionized system and those of a corresponding system with an additional proton [23,24].

In the context of single-determinant approaches for the determination of core-ionized states, a prominent role is played by the ΔSCF (Δ Self-Consistent Field) techniques [25,26,27,28,29,30]. They compute the core excitation energy as the difference between the energy associated with the single Slater determinant wavefunction describing the investigated core ionized state ($\;\Psi^{\prime })$ and the energy corresponding to the single Slater determinant wavefunction for the ground state ($\Psi_{0})$:

(1) $$E_{ion}=E\left(\;\Psi^{\prime }\right)-E\left(\Psi_{0}\right)$$

The strategy at the basis of the ΔSCF methods was initially developed in the framework of the Hartree-Fock (HF) formalism. It was afterwards extended to the Kohn-Sham (KS) scheme of density functional theory (DFT) [31,32] and to the multiconfigurational self-consistent field (MCSCF) approach [17,33]. Moreover, the possibility of introducing dynamical electron correlation at MP2 level has also been considered [33]. 

However, the different types of ΔSCF techniques developed so far do not differentiate for the quantum chemical level of theory with which the computations are performed (e.g., HF or KS-DFT), but rather for the strategy through which the (single Slater determinant) wavefunction of the core-ionized state is determined. A very well-known strategy/algorithm to achieve this goal is the (initial) maximum overlap method (MOM/IMOM) devised by Gill and coworkers [34,35,36,37,38]. In this case, one starts with a set of guess molecular orbitals (MOs) that are obtained by removing a core orbital from the spin-α or spin-β sets (i.e., one starts with a singly occupied core molecular orbital). These initial sets of MOs are then optimized through a modified SCF cycle, in which the occupation of the spin-α and spin-β molecular orbitals at each iteration is not guided by the *aufbau* principle, but by a criterion based on the projections of the current MOs onto the space of the molecular orbitals at the previous iteration (MOM case) [34,35,36] or onto the space of the initial guess MOs (more stable IMOM version) [37,38]. The MOM/IMOM algorithm is certainly one of the simplest ΔSCF approaches, but it also presents some drawbacks and limitations [39]. In fact, in the MOM case, the reference molecular orbitals may deviate from the target *non-aufbau* state. On the other hand, in the IMOM version, convergence difficulties are sometimes observed. To solve these problems, other ΔSCF algorithms have been introduced. Remarkable examples are the combination of the MOM with a direct optimization approach [40,41], the square gradient minimization (SGM) [42] and the state-targeted energy projection (STEP) [43]. In all the above-mentioned ΔSCF variants, the wavefunction of the core-ionized state is always non-orthogonal to the ground state one. To this regard, although Gill and collaborators clearly indicated that the importance of orthogonality is overstated [34], here it is also worth citing single Slater determinant techniques that allow the determination of core-ionized state wavefunctions that are orthogonal to those of the corresponding ground states. They are the local self-consistent field (LSCF) [44,45,46] and asymptotic projection (AP) methods [47,48,49], where the emptied core MO is kept frozen and orthogonal to the occupied molecular orbitals throughout the SCF procedure [12,50,51,52,53,54,55,56]. 

All the ΔSCF approaches in the previous paragraph were cited because they allow the treatment of core-ionized states (which are the main targets of the present work), but it is worth pointing out that they have been developed and can be exploited also for the investigation of excited states. To conclude the overview on the ΔSCF techniques, one should also bear in mind that spin-contamination may affect the results of ΔSCF calculations [39]. This drawback can be partially overcome through an *a posteriori* spin purification by exploiting the spin-projection protocol [39,57]. 

Although all the above-cited single Slater determinant approaches for core-ionized states have a significantly lower computational cost than techniques based on many-determinant wavefunctions, their direct application to very large biosystems is still non-straightforward. In fact, their computational cost approximately increases as $M^{3}$, M being the number of basis functions employed in the calculations. Therefore, as the system size grows, also the computations based on these methods become impractical, if not even impossible.

As of today, the only possible way of routinely applying ΔSCF techniques to macromolecules of biological interest is to couple them with the so-called embedding methods [58]. The latter are strategies where the chemically crucial subunit of the examined system is treated through a high-level quantum chemistry technique, while the rest is described by means of a lower-level approach. A typical example of embedding method is the very popular and pioneering quantum mechanics/molecular mechanics (QM/MM) technique [59,60,61,62]. More recently, this has been followed by the development of fully quantum mechanical embedding strategies, such as the more advanced density matrix [63,64,65,66] and density functional [67,68,69,70,71,72,73,74,75,76,77,78,79,80,81,82] embedding approaches. 

In this framework, it is worth considering that the above-mentioned LSCF and AP methods have already been coupled with molecular mechanics through the traditional QM/MM scheme to determine core-ionized states and core-ionization energies of (relatively) large (bio)molecules (e.g., extended polypeptides, polymers, and even proteins) [12,50,51]. Following these examples, in the present work, we will show that core-ionized states and core-ionization energies can be respectively modeled and evaluated also through the IMOM/ELMO (initial maximum overlap method/extremely localized molecular orbital) technique, a single Slater determinant-based embedding approach recently developed for the treatment of excited states of large systems [83].

The IMOM/ELMO strategy [83] resulted from the coupling of the IMOM [37,38] with the QM/ELMO (quantum mechanics/extremely localized molecular orbital) approach [84,85,86,87,88,89,90]. The latter is a fully quantum mechanical embedding technique, in which the most important region of the system/molecule under exam is treated by means of a traditional quantum chemical method, while the remaining part (i.e., the environment) is described with frozen extremely localized molecular orbitals (ELMOs) [91,92,93] previously transferred from the recently constructed ELMO databanks [94,95,96] or from proper model molecules. Initially proposed in the framework of the restricted Hartree-Fock formalism [84], the QM/ELMO technique has been gradually improved and extended both to more advanced strategies for the treatment of ground states (such as, DFT and different post-HF approaches) [85] and to methods for the investigation of excited states (e.g., time-dependent density functional theory (TDDFT), equation-of-motion coupled cluster (EOM-CC) and IMOM) [83,86]. In all cases (including the IMOM/ELMO method for excited states), it was observed that the QM/ELMO calculations provide results that are in excellent agreement with those resulting from corresponding fully quantum mechanical computations, but with significantly lower computational costs.

In this work, to assess the capabilities of the IMOM/ELMO strategy also for the treatment of core-ionized states, the results of the IMOM/ELMO calculations will be compared to those obtained through fully IMOM ΔSCF computations (when these were feasible). In addition to assessing the performances of the new proposed method on relatively small systems, test calculations were performed by also considering larger molecules, such as large polypeptides and a small protein.

The paper is organized as follows. In the next section, the foundations of the two strategies (IMOM and QM/ELMO) combined in the IMOM/ELMO approach will be reviewed. In Section 3, the investigated systems, and the computations to obtain the analyzed core-ionized states will be described. Afterwards, in Section 4, the collected results will be shown and discussed. Finally, conclusions and future perspectives will be drawn and presented in Section 5.

## 2. Theory

In this section, we will briefly describe the two techniques that are at the basis of the novel IMOM/ELMO approach [83] for the determination of core-ionized states of large molecular systems, namely the initial maximum overlap method [37,38] (Section 2.1) and the quantum mechanics/extremely localized molecular orbital technique [84,85,86,87,88,89,90] (Section 2.2).

### 2.1. Initial Maximum Overlap Method

As in any ΔSCF approach, also in the initial maximum overlap method [37,38], the core-ionized excitation energies are obtained by computing the difference between the energies corresponding to the core-ionized state and the ground state wavefunctions (see Equation (1)). Therefore, the IMOM procedure can be schematized through the following points.

A. *Determination of the ground state wavefunction and energy.* This calculation is generally performed at Hartree-Fock or Kohn-Sham DFT level. This step is fundamental to obtain the starting molecular orbitals, from which one can select those MOs that should work as references in the core-ionized state computation (see points B and C).

B. *Selection of the reference MOs for the core-ionized state computation.* To accomplish this task, one electron is usually removed from one of the core orbitals in the spin-α or spin-β sets of MOs obtained through the ground state calculation (see point A).

C. *Determination of the core-ionized state wavefunction and energy.* Using as references the sets of spin-α and spin-β MOs selected at the previous point, a new Hartree-Fock or Kohn-Sham DFT calculation is performed. However, unlike the traditional SCF procedure, at each iteration of the cycle the spin-α and spin-β MOs are not occupied following the *aufbau* principle (i.e., by considering the lowest energy MOs), but according to an alternative criterion that chooses those molecular orbitals that have the largest projections onto the space of the reference orbitals. The projection $p_{j}$ associated with a generic molecular orbital $\phi_{j}^{new}$ is defined as follows:

(2) $$p_{j}={\left[\;\overset{N_{ref}}{\underset{i=1}{\sum }}{\left(S_{ij}\right)}^{2}\;\right]}^{1/2}$$

with $N_{ref}$ as the number of reference MOs, and $S_{ij}$ as the overlap integral between the new molecular orbital $\phi_{j}^{new}$ at the current iteration and the reference molecular orbital $\phi_{i}^{ref}$. The overlap integral can be also explicitly expressed in this way:

(3) $$S_{ij}=\overset{M}{\underset{\mu =1}{\sum }}\overset{M}{\underset{\nu =1}{\sum }}C_{\mu i}^{ref}\;O_{\mu \nu }\;C_{vj}^{new}$$

where M is the total number of used basis functions, $\left\{C_{\mu i}^{ref}\right\}$ and $\left\{C_{vj}^{new}\right\}$ the coefficients that expand $\phi_{i}^{ref}$ and $\phi_{j}^{new}$ in the chosen basis set, respectively, and $\;O_{\mu \nu }$ the overlap integral between the μ-th and ν-th basis functions.

D. *Computation of the core-ionization energy.* The core-ionization energy is computed according to Equation (1) exploiting the energies obtained from the calculations described at points A and C. As for the excited state case [83], also here the ground and core-ionized state computations are carried out on the same molecular geometry, without introducing any correction due to the zero-point vibration energy. Therefore, only vertical core-ionization energies are calculated. 

The above-described IMOM algorithm was implemented in a locally modified version of the *Gaussian09* quantum chemistry suite of programs [97] by simply adapting the SCF routines. If necessary, correlation energy corrections can be introduced by performing post-Hartree-Fock calculations that exploit the MOs resulting from the SCF computation of the core-ionized state. This is generally done at MP2 level [34,35,36,37,38]. However, as it will be described in the section dedicated to the computational details, in this work we carried out only IMOM computations at KS-DFT level, and, in particular, we always adopted the unrestricted Kohn-Sham (UKS) scheme to treat the open-shell systems for the determination of the core-ionized state wavefunctions and energies. 

### 2.2. QM/ELMO Technique

Before discussing the QM/ELMO algorithm in detail, it is worth stressing that ELMOs are molecular orbitals strictly localized on small molecular subunits (i.e., atoms, bonds, and functional groups) [91,92,93]. Because of this strict localization, ELMOs can be unambiguously associated with molecular fragments and considered as electronic ^®^LEGO building blocks easily transferable from molecule to molecule [94,95,98,99,100,101,102]. To exploit this feature, databanks of extremely localized molecular orbitals have been recently assembled [96]. These libraries currently cover all the possible elementary units of the twenty natural amino acids, and, through the associated *ELMOdb* program [96], they allow almost instantaneous reconstructions of approximate wavefunctions and electron densities of quite large systems (namely, systems ranging from relatively large polypeptides to proteins). The *ELMOdb* program also enables the transfer of customized ELMOs describing fragments of molecules that are not currently included in the databanks (for instance, ELMOs for fragments of ligands involved in protein-ligand complexes). 

As already briefly mentioned in the Introduction, the quantum mechanics/extremely localized molecular orbital (QM/ELMO) method is a fully quantum mechanical multi-scale embedding approach that subdivides the investigated system into two subunits: the active region and the environment [83,84,85,86,87,88,89,90]. The former (known as QM region) is the most important part of the system under exam from the chemical point of view and, for this reason, it is treated at a fully quantum mechanical level through any traditional method of quantum chemistry (e.g., Hartree-Fock, KS-DFT, post-HF strategies, TDDFT, or EOM-CCSD). On the contrary, the latter (known as ELMO region) is the least significant part, which however needs to be properly modelled to correctly embed the chemically crucial subunit; for this reason, it is described at an approximate quantum chemical level through transferred and frozen extremely localized molecular orbitals.

After the subdivision of the system into QM and ELMO regions, the real QM/ELMO algorithm begins. It can be subdivided into three parts: (i) the transfer of the ELMOs to the ELMO subsystem through the *ELMOdb* program (from the above-mentioned ELMO libraries or from suitable model molecules); (ii) preliminary orthogonalizations; and (iii) QM/ELMO self-consistent field cycle. The second and the third parts will be better outlined below, while more details about the transferability of ELMOs are given in the Supplementary Materials. 

The preliminary orthogonalizations entail three different steps: A) the Löwdin orthonormalization of the transferred extremely localized molecular orbitals; B) the orthogonalization of the basis functions centered on the atoms of the QM region against the Löwdin orthonormalized ELMOs; C) the canonical orthogonalization of the basis functions obtained at point (B). The three steps can be summarized through the following matrix transformation:

(4) $$\chi^{\prime }=\chi \;B$$

with χ as the 1×M array of the M starting non-orthogonal basis functions constituting the supermolecular basis set for the whole investigated system, $\chi^{\prime }$ as the $1\times M_{QM}$ array of the final orthonormal basis functions for the QM subsystem (with $M_{QM}\ll M$), and B as an $M\times M_{QM}$ transformation matrix that is crucial in the self-consistent field algorithm that will be described below. The new orthonormal basis set $\chi^{\prime }$ is then used to formally expand the molecular orbitals of the quantum mechanical region. However, due to relation (4), the MOs of the quantum mechanical subunit are actually given by linear combinations of the original non-orthogonal basis functions centered an all the atoms of the examined system (including those belonging to the ELMO region). Interested readers can find more details on the preliminary orthogonalization procedure in the Supplementary Materials or in the seminal papers of the QM/ELMO technique [84,85].

The QM/ELMO self-consistent cycle consists in the following six-step procedure:

A. Construction of the M×M Fock matrix F in the supermolecular basis set χ, with the generic matrix element $F_{\mu \nu }$ given by:

(5) $$\begin{array}{ll} F_{\mu v} & =\langle \chi_{\mu }\left|{\hat{h}}^{\mathrm{core}}\right|\chi_{v}\rangle \\ & +\sum_{\lambda ,\sigma =1}^{M}P_{\lambda \sigma }^{QM}\left[\left(\chi_{\mu }\chi_{v}|\chi_{\sigma }\chi_{\lambda }\right)-\frac{1}{2}x\left(\chi_{\mu }\chi_{\lambda }|\chi_{\sigma }\chi_{\nu }\right)\right]\\ & +\sum_{\lambda ,\sigma \in ELMO}P_{\lambda \sigma }^{ELMO}\left[\left(\chi_{\mu }\chi_{v}|\chi_{\sigma }\chi_{\lambda }\right)?\frac{1}{2}x\left(\chi_{\mu }\chi_{\lambda }|\chi_{\sigma }\chi_{v}\right)\right]\\ & +\langle \chi_{\mu }\left|{\hat{v}}^{XC}\left[P^{QM}+P^{ELMO}\right]\right|\chi_{v}\rangle \end{array}$$

where ${\hat{h}}^{core}$ is the standard one-electron Hamiltonian operator, $P^{QM}$ and $P^{ELMO}$ are the QM and ELMO one-electron reduced density matrices, respectively, $\chi_{\mu }|{\hat{v}}^{XC}\left[P^{\mathrm{QM}}+P^{ELMO}\right]|\chi_{\nu }$ is the generic (μ,ν) element of the exchange-correlation potential matrix (which has to be neglected for HF/ELMO computations), and x is the fraction of exact exchange (which is equal to 1 in the Hartree-Fock case).

B. Transformation of the Fock matrix F to the orthonormal basis set $\chi^{\prime }$ of the QM subsystem by exploiting the transformation matrix B through the following relation:

(6) $$F^{\prime }=B^{†}F\;B$$

C. Diagonalization of the matrix $F^{\prime }$ to obtain the MOs of the QM subunit:

(7) $$F^{\prime }C^{\prime }=C^{\prime }\;E^{\prime }$$

D. Transformation of the MOs resulting from the previous diagonalization to the supermolecular basis set χ exploiting again the transformation matrix B:

(8) $$C=B\;C^{\prime }$$

E. Computation of the QM one-electron density matrix $P^{QM}$ using the MO coefficients resulting from transformation (8):

(9) $$P_{\;\mu \nu }^{QM}=2\overset{N}{\underset{i=1}{\sum }}C_{vi}^{*}C_{\mu i}$$

It is worth noting that, since the molecular orbitals of the quantum mechanical region are actually expanded over the full supermolecular basis set χ (see comment above after Equation (4)), the $P^{QM}$ density matrix is completely full, with non-zero off-diagonal QM-ELMO blocks.

F. Convergence test on density matrix and energy: if convergence is reached, the iterations halt and properties are computed, otherwise the cycle restarts from point A above by updating the Fock matrix F with the current density matrix $P^{QM}$.

The above-described procedure was implemented by modifying an in-house version of the *Gaussian09* quantum chemistry package [97]. Furthermore, it is worth noting that the QM/ELMO SCF algorithm was also slightly changed to implement the IMOM/ELMO approach [83] used in this work. As for the parent IMOM strategy, to decide the occupation of the molecular orbitals associated with the QM region, the traditional *aufbau* principle was substituted with the new criterion that considers the projections onto the space of the starting reference orbitals (see Equation (2)). 

## 3. Computational Details

To evaluate the capabilities of the IMOM/ELMO approach in treating core-ionized states of extended systems, we validated the technique by performing computations on both small and large molecules. To accomplish this task, we initially considered two systems with a relatively long alkyl chain (namely, decane and 2-decanone) and seven small biomimetic molecules (i.e., trans-N-methylformamide, cis-N-methylformamide, N,N-dimethylformamide, acetamide, trans-N-methylacetamide, cis-N-methylacetamide, N,N-dimethylacetamide). Afterwards, we investigated core-ionized states of systems that are more interesting from the biological/biochemical point of view. We started from the isolated alanine amino acid and then, to study the influence of the chemical environment, we gradually increased the size of the investigated molecules by considering the poly-alanine-tripeptide and the larger poly-alanine-pentadecapeptide (both polypeptides in the α-helix and β-sheet conformations). Finally, we assessed the capabilities of the IMOM/ELMO approach on a small protein by evaluating the core-ionization energies for different atoms of the glutamate residue in the 46-residue protein crambin.

The computational details for each of the above-mentioned case-studies will be described in the following subsections. All the calculations here reported were carried out by exploiting the *Gaussian09* quantum chemistry program [97], both in its standard version and in our locally modified variant where the IMOM/ELMO approach [83] was implemented.

### 3.1. Test Calculations of Decane and 2-Decanone

**Figure 1:**
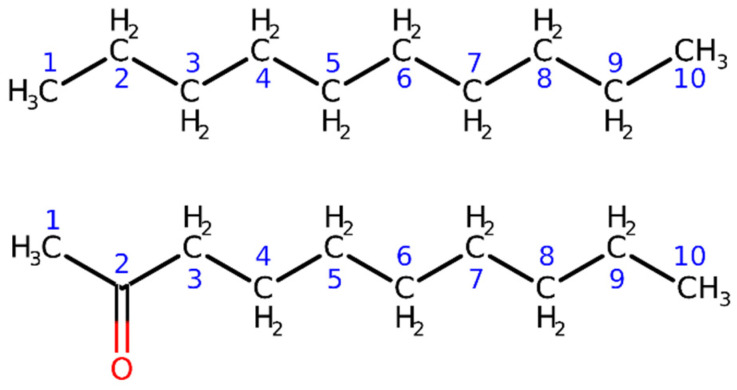
Schematic representation of the model systems considered in the first validation tests on the IMOM/ELMO method for the determination of core-ionized states: decane and 2-decanone (top and bottom panels, respectively). The numbers indicate the labels of the carbon atoms gradually included in the quantum mechanical region of the IMOM/ELMO computations.

As starting points for these preliminary test calculations, we considered the molecular geometries of decane and 2-decanone (see Figure 1) optimized at B3LYP level with the 6-311++G(d,p) set of basis functions. The obtained atomic coordinates were afterwards exploited to perform fully IMOM and IMOM/ELMO computations at DFT level with functionals B3LYP and PBE0, and with different Pople basis sets (6-31G(d), 6-311G(d), 6-31+G(d), and 6-311+G(d)).

For decane, we focused on the 1s core-ionization energy of the carbon atom belonging to one of the two terminal methyl groups. For 2-decanone, we considered the 1s core-ionization energy for the carbonyl carbon of the molecule. As illustrated in the Theory section, to perform both the IMOM and IMOM/ELMO computations on the above-mentioned systems, we initially selected the reference orbitals by analyzing the sets of MOs resulting from the closed-shell calculations for the ground states. To this purpose, for decane we removed the 1s-β molecular orbital mainly localized on the carbon atom of one of the terminal methyl groups; for 2-decanone, we took out the 1s-β molecular orbital mainly localized on the carbonyl carbon. We afterwards carried out UKS computations to determine wavefunctions and energies of the core-ionized systems.

The IMOM/ELMO calculations were performed by progressively increasing the size of the quantum mechanical subunit. This allowed us to evaluate the influence of the ELMO embedding on the treatment of the core-ionized states. In the decane case, we included from three to eight alkyl groups in the QM region; in other words, we considered from three to eight carbon atoms (and the bonded hydrogens) in the QM subsystem. For the 2-decanone molecule, we considered from one to six CH_2_ units along with the terminal CH_3_C=O group in the quantum mechanical subunit; namely we included from three to eight carbons (and the bonded non-carbon atoms) in the QM region. See again Figure 1 for the labels assigned to the different carbon atoms in decane and 2-decanone. The results of the IMOM/ELMO computations (i.e., the values of the core-ionization energies) were compared to those obtained through the corresponding fully IMOM calculations.

### 3.2. Test Calculations of Small Biomimetic Molecules

**Figure 2:**
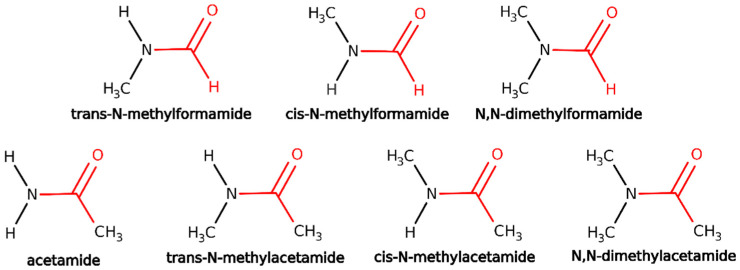
Biomimetic molecules with a peptide bond for the second set of validation tests on the IMOM/ELMO method for the determination of core-ionized states. The QM regions for the performed IMOM/ELMO computations are depicted in red, while the ELMO regions are shown in black.

To further validate the IMOM/ELMO method for the treatment of core-ionized states, we also considered the seven above-mentioned small biomimetic molecules characterized by the presence of a peptide bond (see Figure 2). Also in this case, the geometries of the investigated systems were preliminarily optimized at B3LYP/6-311++G(d,p) level. Afterwards, exploiting the obtained atomic coordinates, fully IMOM and IMOM/ELMO computations were performed using the PBE0 functional along with the 6-311++G(d,p) basis set.

For each analyzed molecule, we determined the 1s core-ionization energy for the carbon and oxygen atoms of the carbonyl group in the peptide bond. For the IMOM/ELMO computations, the QM subunits consisted of the whole C=O group, the nearest neighbor C-N bond, the nearest neighbor C-H/C-C bond (for formamides/acetamides, respectively), and the nitrogen lone-pair electrons delocalized over the peptide bond (see the regions depicted in red in Figure 2). Additionally, for all the test calculations described in this paragraph, the reference orbitals were selected by removing the 1s-β MOs mainly localized on the carbonyl carbons/oxygens and identified through the analysis of the sets of molecular orbitals obtained through the preliminary closed-shell computations carried out on the non-ionized molecules. UKS calculations were afterwards performed to treat the resulting open-shell systems. The core-ionization energies obtained at IMOM/ELMO level were compared to the corresponding fully IMOM values.

### 3.3. Test Calculations on Alanine Polypeptides

**Figure 3:**
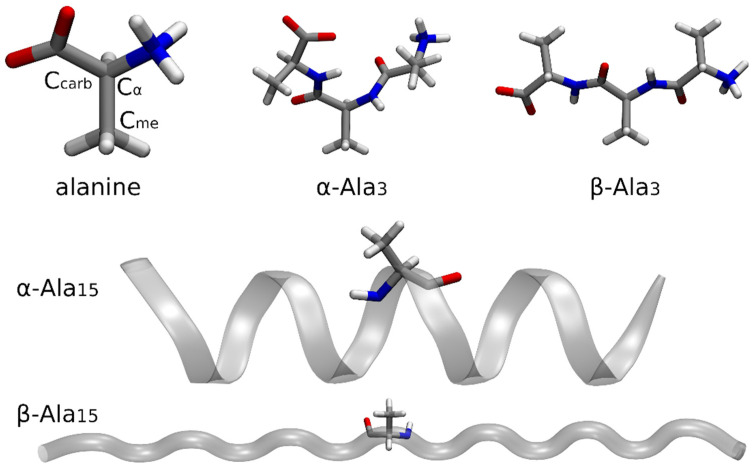
Optimized structures of the isolated alanine amino acid, poly-alanine-tripeptide (α-helix- and β -sheet-like geometries), and poly-alanine-pentadecapepide (α -helix and β -sheet conformations) in their zwitterionic forms. For the sake of clarity, the carbon atoms for which the 1s core-ionization energies were computed are explicitly indicated only for the isolated alanine molecule.

To start evaluating the proposed strategy on systems that are more significant from the biological point of view, we afterwards examined the isolated alanine amino acid and different alanine polypeptides (namely, the poly-alanine-tripeptide, Ala_3_, and the poly-alanine-pentadecapeptide, Ala_15_). A graphical representation of the investigated molecules is given in Figure 3.

All the systems were considered in their zwitterionic forms. The geometry of the isolated alanine amino acid was preliminarily optimized at B3LYP/6-311++G(d,p) level. The same level of theory and basis set were also used to optimize the α-helix- and β-sheet-like conformers of the Ala_3_ polypeptide. Concerning Ala_15_, its α-helix and β-sheet conformations were optimized at semiempirical PM6 level in implicit solvent (water), with constraints on the backbone atoms to avoid folding in the β-sheet case.

For the isolated alanine amino acid, we performed fully IMOM calculations on the whole system at PBE0/6-311G(d,p) level to determine the 1s core-ionization energies of carbon atoms $C_{\alpha }$, $C_{\mathrm{carb}}$, and $C_{\mathrm{met}}$ (see again Figure 3). We then carried out IMOM/ELMO computations on Ala_3_ and Ala_15_ in both their conformations. In those cases, the QM region was treated at DFT level with the PBE0 functional, while the 6-311G(d,p) set of basis functions was used for both the QM and ELMO subunits. Through these IMOM/ELMO calculations we obtained the 1s core-ionization energies of carbon atoms $C_{\alpha }$, $C_{\mathrm{carb}}$, and $C_{\mathrm{met}}$ in the central residues of the investigated polypeptides (i.e., Ala2 in Ala_3_ and Ala7 in Ala_15_). For this reason, in all the performed IMOM/ELMO computations, the adopted quantum mechanical regions practically coincided with the central residues. 

The results of the IMOM/ELMO calculations on the polypeptides were compared to those obtained through the fully IMOM computations on the isolated alanine amino acid to evaluate the effects of the chemical environment.

### 3.4. Application to the Protein Crambin

The final test computations were carried out on the protein crambin, for which we considered its 0.54 Å high-resolution crystal structure (PDB code: 1EJG). Starting from the PDB file, we initially kept only the atoms belonging to the major components in the disordered parts of the protein and, after properly defining the protonation states of the different residues, we added the coordinates of the missing hydrogen atoms by exploiting the *tleap* utility of the *AMBER* Molecular Dynamics package [103].

**Figure 4:**
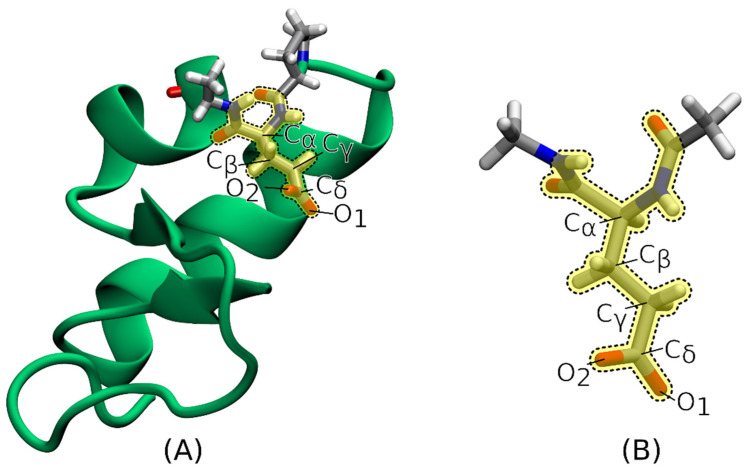
(**A**) Glutamate residue and its neighboring atoms (both depicted in licorice representation) in the protein crambin (mainly depicted in cartoon representation), with the labels indicating the glutamate atoms for which the 1s core-ionization energies were computed at IMOM/ELMO level; (**B**) reduced model system used for the IMOM/ELMO and fully IMOM calculations performed on the isolated glutamate residue. In both cases the QM region is highlighted in yellow and framed by a dotted line.

Using the geometry resulting from the above-described procedure, we determined the 1s core-ionization energies of six atoms belonging to the glutamate residue of the protein: the $C_{\alpha }$ atom and the five non-hydrogen atoms of the side chain (see Figure 4A). To this purpose, we carried out IMOM/ELMO calculations with only the glutamate residue corresponding to the QM region (see again Figure 4A), which was treated at DFT level with the PBE0 functional. The 6-311G(d,p) basis set was exploited for the whole system.

We also performed IMOM/ELMO and fully IMOM computations (with the same level of theory and the same basis set indicated above) on the isolated glutamate residue (geometry extracted from the protein structure), properly saturated with the N-methyl amino (CH_3_-NH-) and acetyl (CH_3_-CO-) groups (see Figure 4B) using the *ProScrs.py* utility provided within the *AMBER* suite of programs [103]. In the case of the IMOM/ELMO calculations, we adopted a QM region identical to the one used in the IMOM/ELMO computations on the whole protein, while the ELMO subunit practically reduced to the above-specified saturating terminal groups.

The comparison of the 1s core-ionization energies obtained through the calculations on the whole and reduced systems allowed us to assess the effects of the chemical environment in the protein.

### 3.5. ELMO Calculations and Transfer

The extremely localized molecular orbitals employed in the IMOM/ELMO computations described in the previous subsections were taken from the ELMO databanks or obtained by means of calculations carried out on suitable model molecules by using a modified version of the *GAMESS-UK* suite of programs [104] where the Stoll equations [91,92] were implemented (more details about the ELMO theory are given in the Supplementary Materials).

Pertaining to decane and 2-decanone, the ELMOs for the alkyl groups were computed on the butane molecule using the different basis sets considered in the test calculations described in Section 3.1 and exploiting a geometry optimized at B3LYP/cc-pVDZ level. Concerning the small biomimetic molecules, the required extremely localized molecular orbitals were computed with the 6-311++G(d,p) basis set on methylamine, acetamide and acetaldehyde, whose geometries were previously optimized at B3LYP/6-311++G(d,p) level. For the alanine polypeptides, the ELMOs were properly transferred from the ELMO database for the 6-311G(d,p) set of basis functions. Finally, pertaining to the investigation on the protein crambin, the extremely localized molecular orbitals for the protein residues were directly taken from the ELMO libraries (6-311G(d,p) basis set), while those describing the terminal N-methyl amino and acetyl groups resulted from calculations carried out with the 6-311G(d,p) set of basis functions on the N-methylacetamide molecule exploiting a geometry previously optimized at B3LYP/cc-pVDZ level.

All the transfers of ELMOs in this work were conducted by means of the *ELMOdb* program [96], which uses the technique devised by Philipp and Friesner to rotate strictly localized bond orbitals [94,105].

## 4. Results and Discussion

In this section we will show and analyze the results of the test calculations described above. First, we will focus on the preliminary validation tests on small systems: decane and 2-decanone (Section 4.1), and the small biomimetic molecules with a peptide bond (Section 4.2). Afterwards, we will inspect and discuss the results obtained for larger biomolecules with the main goal of start investigating environmental effects in biological molecules: alanine polypeptides (Section 4.3), and the protein crambin (Section 4.4).

### 4.1. Decane and 2-Decanone

**Figure 5:**
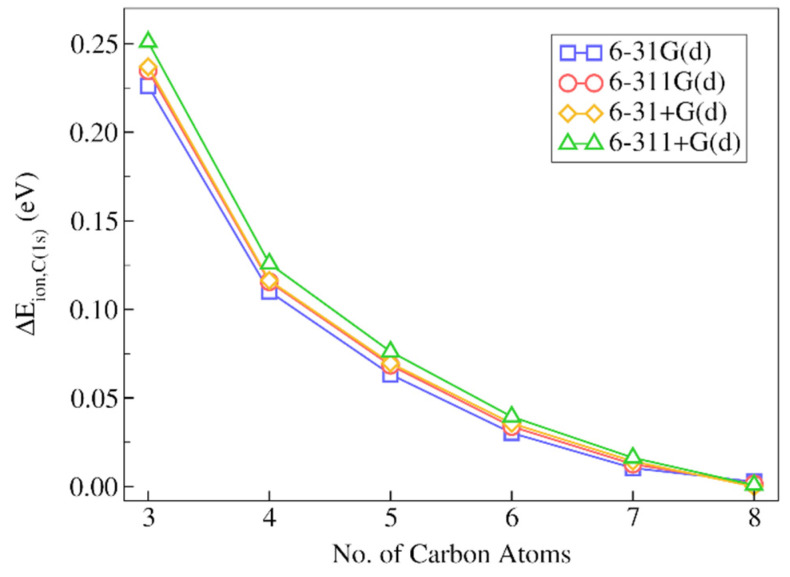
Absolute deviations of the IMOM/ELMO 1s core-ionization energies for the terminal carbon atom of decane from the reference fully IMOM values (${\Delta E}_{\mathrm{ion},C(1s)}$), as a function of the number of carbon atoms gradually included in the quantum mechanical region of the QM/ELMO calculations. Only the results of the computations performed with the PBE0 functional are reported.

First, let us consider the results obtained for decane using the PBE0 functional with the four Pople basis sets considered in our calculations (see Section 3.1). In Figure 5, we showed the absolute differences between the IMOM/ELMO and fully IMOM 1s core-ionization energies for the terminal carbon atom, as a function of the number of carbons gradually included in the QM region for the performed QM/ELMO computations (see also Table S1 in the Supplementary Materials for the actual data). In Table 1, we also reported the corresponding relative discrepancies of the IMOM/ELMO values.

By analyzing the results, we can observe that, already when only three carbon atoms are included in the quantum mechanical subsystem, the IMOM/ELMO core-ionization energies are in very good agreement with the results of the fully IMOM computations, with absolute discrepancies in the 0.226–0.251 eV range, and relative absolute deviations lower than 0.1%. The situation further improves by treating more alkyl groups at fully QM level. In fact, with only one additional CH_2_ group in the QM subunit, the absolute discrepancies are barely above 0.1 eV for all basis sets (0.110–0.126 eV range) with relative errors of about 0.04%. The description is better and better as we increase the QM region size and, as one should expect, the IMOM/ELMO results approach the fully IMOM ones.

Concerning the basis set dependence, we can clearly see that the results are practically identical for all the sets of basis functions used in our investigation. By inspecting the results more in detail, we can only highlight the fact that the computations performed with basis sets without diffuse functions almost always provided lower discrepancies, with the lowest ones generally obtained through the 6-31G(d) basis set.

The results of the calculations carried with the B3LYP functional are analogous to those described above with the functional PBE0. For the sake of completeness, they are reported in the Supplementary Materials (see Figure S3, and Tables S2 and S3). 

**Figure 6:**
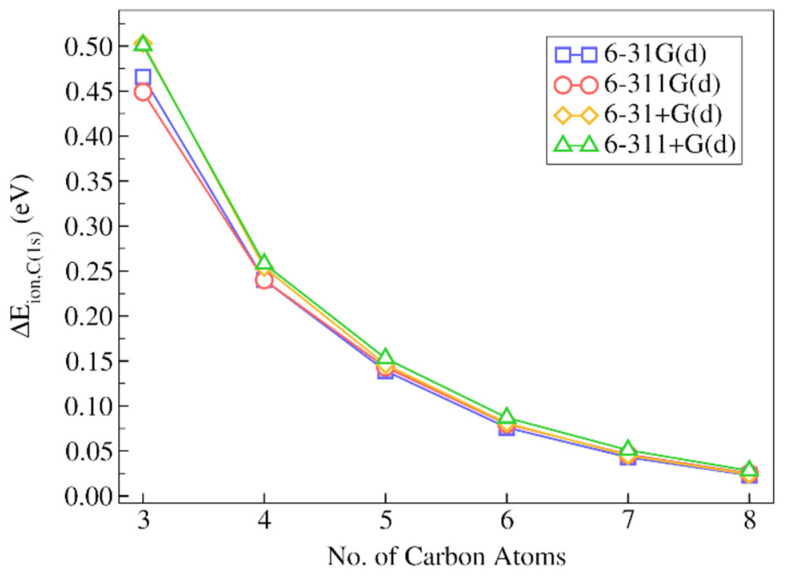
Absolute deviations of the IMOM/ELMO 1s core-ionization energies for the carbonyl carbon atom of 2-decanone from the reference fully IMOM values (${\Delta E}_{\mathrm{ion},C(1s)}$), as a function of the number of carbon atoms gradually included in the quantum mechanical region of the QM/ELMO calculations. Only the results of the computations performed with the PBE0 functional are reported.

Now, let us analyze the results for the 2-decanone molecule. In Figure 6 we depicted the absolute discrepancies of the IMOM/ELMO 1s core-ionization energies for the carbonyl carbon from the corresponding reference fully IMOM results as a function of the number of carbons included in the QM subunit. The actual values of the absolute deviations are given in Table S4 of the Supplementary Materials, while the relative discrepancies are reported in Table 2.

The general trends are analogous to those observed for decane, although it is worth observing that, in this case, the discrepancies are slightly larger for equal numbers of carbon atoms in the quantum mechanical subunit. This may be explained by considering two different aspects. The first one consists in the fact that, for equal number of carbons included in the QM subunit, the atom for which the 1s core-ionization energy is computed (i.e., C2 in the bottom panel of Figure 1) is always closer to the ELMO region compared to the case of decane, for which the atom of interest is C1 (see the top panel of Figure 1). The second aspect is related to the extremely localized molecular orbitals used to describe the chemical environment; in fact, as mentioned in Section 3.5, for both the computations on decane and the calculations on 2-decanone, the transferred ELMOs were determined on butane, which is clearly a more suitable model molecule to describe the alkyl chain of decane.

Anyway, analyzing in detail the results reported in Figure 6 and Table 2, we can still observe that the absolute discrepancies are quite small, already when only three carbon atoms are treated at fully quantum mechanical level (absolute discrepancies in the 0.473–0.510 eV range), with relative deviations between 0.15% and 0.18%. The IMOM/ELMO description is already much better by adding only one single CH_2_ moiety (i.e., with four carbon atoms overall) in the QM subunit. In fact, the relative discrepancies drop below 0.1% with absolute deviations between 0.251 and 0.269 eV. As for decane, and as one should expect from QM/ELMO calculations, the IMOM/ELMO results approach the fully IMOM ones as the QM region size increases. For example, we can observe that the absolute deviations start being lower than 0.1 eV for all the considered basis sets when at least six carbon atoms belong to the quantum mechanical subsystem.

As observed above for decane, it is also possible to notice that the trends are practically identical for all the sets of basis functions used in the computations. However, also in this situation, the discrepancies are almost always lower for calculations performed with basis sets without diffuse functions. The best results are again obtained with the 6-31G(d) basis set.

Finally, we also considered the outcomes of the computations carried out with functional B3LYP. By inspecting Figure S4 and Tables S5 and S6 in the Supplementary Materials, we can notice trends completely analogous to those observed at PBE0 level.

Based on the results presented and discussed above, it is evident that the IMOM/ELMO approach is successful in obtaining quite accurate core-ionization energies by treating only a small part of the investigated system at a fully quantum chemical level. Therefore, although further tests on biomimetic molecules still have to be discussed (see Section 4.2), the points highlighted in this subsection would already justify the application of the proposed embedding technique to larger systems of biological interest (see Section 4.3 and Section 4.4).

### 4.2. Small Biomimetic Molecules

Concerning the test calculations performed on the small biomimetic molecules, the IMOM/ELMO results are shown in Table 3, where we have reported the absolute and relative deviations with respect to the reference fully IMOM values for the 1s-core ionization energies of the carbonyl carbon and carbonyl oxygen atoms involved in peptide bonds.

From the inspection of the results, we can immediately see that the discrepancies are generally larger than those observed for the test calculations reported in Section 4.1. This is particularly evident for the 1s core-ionization energies of the carbonyl carbon atoms, for which the absolute deviations are larger than or very close to 1 eV and the relative discrepancies fall in the 0.30–0.55% range. However, the IMOM/ELMO description is significantly better for the 1s core-ionization energies of the carbonyl oxygen atoms. In fact, in those cases, the absolute deviations are almost always lower than 1 eV, with relative discrepancies ranging from 0.11% to 0.20%.

The better performance of the IMOM/ELMO technique in the determination of the 1s core-ionization energies for the carbonyl oxygens can be explained with the fact that, in those situations, we used larger “QM buffer regions” around the atoms of interest (see Figure 2 and computational details in Section 3.2). On the contrary, for the IMOM/ELMO computations of the 1s core-ionization energies associated with the carbonyl carbons, the atom under examination was always too close to the boundaries with the ELMO regions, thus leading to quite large discrepancies compared to the fully quantum mechanical reference values. 

Based on the previous considerations, we can also explain the reasons why we observed smaller deviations for the test calculations carried out on decane and 2-decanone (see Section 4.1). In those cases, almost all the QM regions were larger than those adopted for the computations discussed in this subsection. Only the smallest QM subsystem for the IMOM/ELMO computations performed on 2-decanone provided a “QM buffer” for the examined atom that is comparable to those adopted for the carbonyl oxygens in the calculations on the biomimetic molecules. In fact, the relative discrepancies reported in Table 3 for the carbonyl oxygen atoms are almost always comparable to those obtained for the carbonyl carbon of 2-decanone when only three carbon atoms were included in the QM region (see Table 2).

Therefore, the reported results indicate the importance of using sufficiently large quantum mechanical environments around the atoms for which one wants to determine core-ionized states through the IMOM/ELMO technique.

### 4.3. Alanine Polypeptides

The IMOM/ELMO results for the alanine polypeptides are reported in Table 4, where we also show the absolute discrepancies of the 1s core-ionization energies with respect to the values obtained for the isolated alanine amino acid.

First, let us focus on the molecules in the α-helix conformation. By considering the results for Ala_3_, we can observe that the 1s core-ionization energies of the considered carbon atoms do not change significantly with respect to the values obtained for the isolated alanine. The absolute deviations are never larger than 0.26 eV in absolute value, with relative discrepancies always lower than 0.1%. Pertaining to the Ala_15_ polypeptide, although all the variations are still negligible for the carbon atoms in the central residue (notably, they are lower than 0.76 eV in absolute value), they are slightly larger for $C_{\alpha }$ and $C_{\mathrm{met}}$. In fact, in those cases, the relative deviations with respect to the values for the isolated alanine become larger than 0.2% in absolute value. On the other hand, the variation of the 1s core-ionization energy for $C_{\mathrm{carb}}$ slightly decreases from 0.24 eV to 0.18 eV by passing from Ala_3_ to Ala_15_.

The general trend is analogous for the molecules in the β-sheet conformation. In fact, for the three carbon atoms examined in the poly-alanine-tripeptide, the deviations with respect to the values obtained for the isolated amino acid never exceed 0.57 eV in absolute value, corresponding to relative discrepancies that are smaller than 0.2%. As for the α-helix conformation, also in this situation the deviations increase in the poly-alanine-pentadecapeptide (in this case, more remarkably for $C_{\mathrm{carb}}$). However, as above, they remain quite small, namely, they are never larger than 0.91 eV, with a maximum relative discrepancy of about 0.3% in absolute value.

The above-described results clearly indicate that, for the investigated systems, the effects of the environment are negligible, which can be explained with the fact that the ground state of the examined molecules is neutral and only slightly polarized/influenced by the embedding of the transferred extremely localized molecular orbitals. In this context, it is also worth noting that the reported trends are fully consistent with those already observed by Loos and Assfeld in an analogous study, where almost the same systems were studied by applying the multi-scale embedding LSCF/MM approach (in that case non-zwitterionic systems were considered) [50].

### 4.4. Protein Crambin

The numerical results obtained for the protein crambin are shown in Table 5, where we reported the 1s core-ionization energies for the carbon $C_{\alpha }$ and the side-chain non-hydrogen atoms of residue Glu23, as resulting from the different types of calculations described in SubSection 3.4.

Unlike what was observed for the alanine polypeptides, the validation tests discussed in this subsection indicate that the environment does indeed have an influence on the 1s core-ionization energies of the carbon and oxygen atoms of the glutamate residue in the protein crambin. In fact, by comparing the results of the IMOM/ELMO calculations on the full protein to those of the IMOM/ELMO computations on the isolated residue, we can notice that all the ionization energies significantly increase. In fact, all the variations are larger than 4.75 eV, with the largest one amounting to 5.01 eV and corresponding to the carbon $C_{\alpha }$ atom. These quite large chemical shifts can be interpreted as due to the non-negligible interactions of the overall negative charge of the glutamate residue with the rest of the protein. They are in line with those already observed by Ferré and Assfeld [12], who carried out ΔSCF computations on the same system by exploiting their multiscale LSCF/MM approach (but on a different geometry and using a different quantum chemical level of theory).

Finally, to prove that the obtained chemical shifts were not the results of the adopted QM/ELMO model, and particularly of the ELMOs used to describe the frontiers bonds between the QM and ELMO subunits, we also carried out an additional series of fully-IMOM computations on the single glutamate residue saturated with the N-methyl amino and acetyl terminal groups. We can notice that the 1s core-ionization energies obtained through these fully IMOM calculations on Glu23 are very close to the corresponding IMOM/ELMO results, with the largest discrepancy amounting only to 0.13 eV in absolute value and corresponding to the $C_{\alpha }$ carbon atom. It is worth noting that this atom is the closest to the frontier between the QM and ELMO regions and, therefore, it is the most affected by the approximation introduced through the QM/ELMO model. However, even in the $C_{\alpha }$ case, the difference between the results of the fully IMOM and IMOM/ELMO calculations on the isolated glutamate residue does not overturn the large chemical shift due to the chemical environment that was observed by means of the IMOM/ELMO computation on the full protein. Therefore, based on the collected results, we can fully trust the outcomes of the IMOM/ELMO calculations and confirm that the observed shifts in the 1s core-ionization energies of the non-hydrogen atoms of Glu23 are due to the environmental interactions with the surrounding residues of the protein crambin.

## 5. Conclusions and Perspectives

In this work, we have considered the possibility of investigating core-ionized states of large (bio)systems through the recently developed IMOM/ELMO embedding technique. The motivation of this study stems from the fact that, although less computationally expensive, even the single Slater determinant-based approaches are not easily applicable to macromolecules of biological interest to determine core-ionized states and the corresponding ionization energies. To overcome this problem, the IMOM/ELMO method treats only the chemically crucial region of the investigated system through the fully quantum mechanical ΔSCF-IMOM algorithm, while the remaining part is approximately described by means of transferred and frozen extremely localized molecular orbitals.

Preliminary test calculations performed on small systems have clearly shown that the proposed embedding strategy is able to provide reliable results. In fact, the IMOM/ELMO computations gave quite accurate core ionization energies (i.e., core-ionization energies in very good agreement with those resulting from traditional IMOM calculations on the whole systems), by treating only a small part of the examined molecules at fully quantum mechanical level. Moreover, the validation tests have also indicated that it is necessary to adopt a sufficiently large “quantum mechanical buffer” around the atom of interest to obtain more and more reliable results. 

The IMOM/ELMO technique has been afterwards successfully applied to larger systems of biological interest (particularly, large polypeptides and a protein). The obtained trends are in full agreement with those resulting from previous investigations that exploited alternative ΔSCF approaches (e.g., the LSCF/MM strategy). This further confirmed the reliability of the proposed embedding method in treating core-ionized states in large biomolecules, especially to account for the surrounding chemical environment of regions that are important from the chemical point of view.

Given the obtained results, we envisage further applications of the new IMOM/ELMO technique, especially with the goal of interpreting or predicting X-ray absorption spectra of large biological macromolecules.

## Figures and Tables

**Table 1:** Relative discrepancies (in %) of the IMOM/ELMO 1s core-ionization energies for the terminal carbon atom of decane with respect to the reference fully IMOM values, as a function of the number of carbon atoms progressively included in the quantum mechanical subsystem of the performed QM/ELMO computations. Only the results of the calculations performed with the PBE0 functional are reported.

No. of Carbon Atoms	IMOM/ELMO Relative Discrepancies for Core-Ionization Energies (%)			
	6-31G(d)	6-311G(d)	6-31+G(d)	6-311+G(d)
3	0.078	0.081	0.081	0.087
4	0.038	0.040	0.040	0.043
5	0.022	0.024	0.024	0.026
6	0.010	0.012	0.012	0.014
7	0.004	0.004	0.005	0.006
8	−0.001	−0.001	0.000	0.000

**Table 2:** Relative discrepancies (in %) of the IMOM/ELMO 1s core-ionization energies for the carbonyl carbon atom of 2-decanone with respect to the reference fully IMOM values, as a function of the number of carbon atoms progressively included in the quantum mechanical subsystem of the performed QM/ELMO computations. Only the results of the calculations performed with the PBE0 functional are reported.

No. of Carbon Atoms	IMOM/ELMO Relative Discrepancies for Core-Ionization Energies (%)			
	6-31G(d)	6-311G(d)	6-31+G(d)	6-311+G(d)
3	0.161	0.157	0.174	0.174
4	0.085	0.086	0.091	0.092
5	0.050	0.051	0.053	0.055
6	0.028	0.029	0.030	0.032
7	0.016	0.017	0.017	0.019
8	0.009	0.010	0.010	0.011

**Table 3:** Absolute and relative discrepancies of the IMOM/ELMO 1s core-ionization energies for the carbonyl carbon and carbonyl oxygen atoms involved in the peptide bonds of the analyzed biomimetic molecules, always with respect to the corresponding fully IMOM reference values (also reported in the table). The results were obtained at PBE0/6-311++G(d,p) level.

Molecule	Carbonyl Carbon			Carbonyl Oxygen		
	Fully IMOM Value (eV)	Absolute Discrepancy (eV)	Relative Discrepancy (%)	Fully IMOM Value (eV)	Absolute Discrepancy (eV)	Relative Discrepancy (%)
Trans-N-methylformamide	293.57	0.88	0.299	536.30	0.63	0.117
Cis-N-methylformamide	293.54	0.89	0.304	536.32	0.60	0.113
N,N-dimethylformamide	293.17	1.25	0.426	536.03	0.83	0.155
Acetamide	293.78	0.72	0.245	536.20	0.61	0.114
Trans-N-methylacetamide	293.35	1.27	0.433	535.88	0.90	0.168
Cis-N-methylacetamide	293.36	1.22	0.415	535.86	0.93	0.173
N,N-dimethylacetamide	293.06	1.61	0.549	535.62	1.09	0.204

**Table 4:** 1s core-ionization energies obtained for carbon atoms $C_{\alpha }$, $C_{\mathrm{carb}}$, and $C_{\mathrm{met}}$ in the isolated alanine amino acid (fully IMOM calculations), in residue Ala2 of polypeptide Ala_3_ (IMOM/ELMO computations) and in residue Ala7 of polypeptide Ala_15_ (IMOM/ELMO calculations). All the computations were performed at PBE0/6-311G(d,p) level. The polypeptides Ala_3_ and Ala_15_ were considered in the α-helix and β-sheet conformations. The absolute discrepancies with respect to the 1s core-ionization energies for the isolated alanine amino acid (which are reported along with the values for the polypeptides in the α-helix conformation for the sake of simplicity) are also given.

Conformation/Atom	1s Core-Ionization Energy (eV)			Absolute Discrepancy (eV)	
	Ala	Ala_3_	Ala_15_	Ala_3_	Ala_15_
α-helix					
$C_{\alpha }$	292.41	292.28	291.65	−0.13	−0.76
$C_{\mathrm{carb}}$	292.79	293.03	292.97	0.24	0.18
$C_{\mathrm{met}}$	291.25	290.99	290.58	−0.26	−0.67
β-sheet					
$C_{\alpha }$		292.28	292.07	−0.13	−0.34
$C_{\mathrm{carb}}$		293.36	293.70	0.57	0.91
$C_{\mathrm{met}}$		290.71	290.59	−0.54	−0.66

**Table 5:** 1s core-ionization energies obtained for the $C_{\alpha }$ and the side-chain non-hydrogen atoms of residue Glu23 in the protein crambin, as resulting from IMOM/ELMO calculations on the full protein (Glu23 as QM region), and from IMOM/ELMO and fully IMOM computations on the isolated glutamate residue properly capped with the N-methyl amino and acetyl terminal groups. All the calculations were carried out at PBE0/6-311G(d,p) level. The deviations from the 1s core ionization energies obtained at IMOM/ELMO level on the isolated residue are also shown.

Atom	1s Core-Ionization Energies (eV)			Absolute Deviations with Respect to IMOM/ELMO (Glu23) (eV)	
	IMOM/ELMO (Crambin)	IMOM/ELMO (Glu23)	Fully IMOM (Glu23)	IMOM/ELMO (Crambin)	Fully IMOM (Glu23)
$C_{\alpha }$	293.38	288.37	288.24	5.01	−0.13
$C_{\beta }$	291.47	286.62	286.60	4.85	−0.02
$C_{\gamma }$	290.71	285.81	285.81	4.90	0.00
$C_{\delta }$	292.22	287.42	287.43	4.80	0.01
O1	534.52	529.72	529.73	4.80	0.01
O2	534.17	529.41	529.41	4.76	0.00

## Data Availability

The data presented in this study are available on request from the corresponding authors.

## Supplementary Materials

The following supporting information can be downloaded at: https://www.mdpi.com/article/10.3390/molecules28010136/s1: overview on theory, transferability, and libraries of extremely localized molecular orbitals (containing Figure S1 with examples of ELMOs and Figure S2 schematically depicting reference frames and atomic triads needed for the ELMO rotations); theoretical details on the preliminary orthogonalization procedure of the QM/ELMO method; Figures S3 and S4 showing the absolute deviations of the IMOM/ELMO 1s core-ionization energies for the terminal/carbonyl carbon of decane/2-decanone from the reference fully IMOM values, as a function of the number of carbon atoms gradually included in the quantum mechanical subunit of the QM/ELMO calculations (results for the B3LYP functional); Tables S1 and S2 reporting the absolute deviations of the IMOM/ELMO 1s core-ionization energies for the terminal carbon of decane from the corresponding fully IMOM values, for different sizes of the QM region adopted in the QM/ELMO computations (Table S1: PBE0 functional; Table S2: B3LYP functional); Table S3 showing the relative discrepancies of the IMOM/ELMO 1s core-ionization energies for the terminal carbon of decane with respect to the reference fully IMOM values, for different sizes of the QM region adopted in the QM/ELMO calculations (results for the B3LYP functional); Tables S4 and S5 reporting the absolute deviations of the IMOM/ELMO 1s core-ionization energies for the carbonyl carbon of 2-decanone from the corresponding fully IMOM values, for different sizes of the QM region adopted in the QM/ELMO computations (Table S4: PBE0 functional; Table S5: B3LYP functional); Table S6 showing the relative discrepancies of the IMOM/ELMO 1s core-ionization energies for the carbonyl carbon of 2-decanone with respect to the reference fully IMOM values, for different sizes of the QM region adopted in the QM/ELMO calculations (results for the B3LYP functional). Citations: [45,84,85,91,93,94,95,96,105].
