# Supplementary material for: Initial Maximum Overlap Method Embedded with Extremely Localized Molecular Orbitals for Core-Ionized States of Large Systems
Source: Molecules. 2022 Dec 24;28(1):136. doi: 10.3390/molecules28010136 (PMC9822432; doi:10.3390/molecules28010136)
Supplement: Supplementary file 1 [file molecules-28-00136-s001.zip › molecules-2098061-supplementary.pdf]

Supplementary Materials for:

Initial Maximum Overlap Method Embedded with Extremely Localized Molecular Orbitals for Core-Ionized States of Large Systems

Giovanni Macetti[(1)*], Alessandro Genoni[(1)*]

(1) Université de Lorraine & CNRS, Laboratoire de Physique et Chimie Théoriques (LPCT), UMR CNRS 7019, 1 Boulevard Arago, F-57078 Metz, France.

* Correspondence to:

- Alessandro Genoni, Université de Lorraine & CNRS, Laboratoire de Physique et Chimie Théoriques (LPCT), UMR CNRS 7019, 1 Boulevard Arago, F-57078 Metz, France. E-mail: alessandro.genoni@univ-lorraine.fr.
- Giovanni Macetti, Université de Lorraine & CNRS, Laboratoire de Physique et Chimie Théoriques (LPCT), UMR CNRS 7019, 1 Boulevard Arago, F-57078 Metz, France. E-mail: giovanni.macetti@gmail.com.

S1. Overview on theory, transferability, and libraries of Extremely Localized Molecular Orbitals

S1.1 Theory of Extremely Localized Molecular Orbitals

ELMOs (Extremely Localized Molecular Orbitals) are molecular orbitals strictly localized on small molecular fragments. They can be calculated through the *a priori* definition of a localization scheme that allows the partitioning of an investigated system into different subunits according to the chemical intuition (in most of the cases, according to the Lewis structure of the examined molecule).[1,2] This fragmentation leads to have a local basis set $\beta_i = \{|\chi_{i\mu}\rangle\}_{\mu=1}^{M_i}$ for each fragment. These local basis sets are constituted only by those basis functions centered on to the atoms belonging to the subunits and are used to expand the ELMOs corresponding to the different fragments. For this reason, the generic ω-th ELMO for the i-th fragment can be written as follows:

$$|\varphi_{i\omega}\rangle = \sum_{\mu=1}^{M_i} C_{i\mu,i\omega} |\chi_{i\mu}\rangle \qquad (S1)$$

Considering the method proposed by Stoll,[1] the system under exam can be described through a single Slater determinant assembled with the ELMOs defined by equation (S1), to which we will hereinafter refer as *ELMO wave function*:

$$|\Psi_{ELMO}\rangle = \frac{1}{\sqrt{(2N)!\,det[\mathbf{S}]}} \hat{A}\left[\prod_{i=1}^{f}\prod_{\omega=1}^{n_i} \varphi_{i\omega}\,\bar{\varphi}_{i\omega}\right] \qquad (S2).$$

In equation (S2), $\hat{A}$ is the usual antisymmetrizer, n_i the number of occupied ELMOs for the i-th fragment, $\varphi_{i\omega}$ a spinorbital with spatial part $\varphi_{i\omega}$ and spin part α, and $\bar{\varphi}_{i\omega}$ a spinorbital with spatial part $\varphi_{i\omega}$ and spin part β, while $det[\mathbf{S}]$ is the determinant of the overlap matrix of the occupied ELMOs (due to the ELMOs non-orthogonality).

ELMOs are computed by minimizing the energy corresponding to the *ELMO wave function* with respect to the coefficients $\{C_{i\mu,i\omega}\}$ in expansion (S1). This is equivalent to solving a set of modified Hartree-Fock equations for each fragment (also known as Stoll equations):[1]

$$\hat{F}_i|\varphi_{i\omega}\rangle = \varepsilon_{i\omega}\,|\varphi_{i\omega}\rangle \qquad (S3)$$

with $\hat{F}_i$ as the modified Fock operator corresponding to the i-th subunit:

$$\hat{F}_i = \left(1 - \hat{\rho} + \hat{\rho}_i^{\dagger}\right)\hat{F}\left(1 - \hat{\rho} + \hat{\rho}_i\right) \qquad (S4),$$

In the previous equation, $\hat{F}$ is the traditional Fock operator, $\hat{\rho}$ the global density operator (which depends on all the occupied ELMOs of the system), and $\hat{\rho}_i$ the density operator for the i-th subunit (which depends only on the occupied ELMOs of the fragment).

As an example, in Figure S1 we depicted the ELMOs that we obtained for the simple water molecule when we defined a localization scheme corresponding to the Lewis structure of the system.

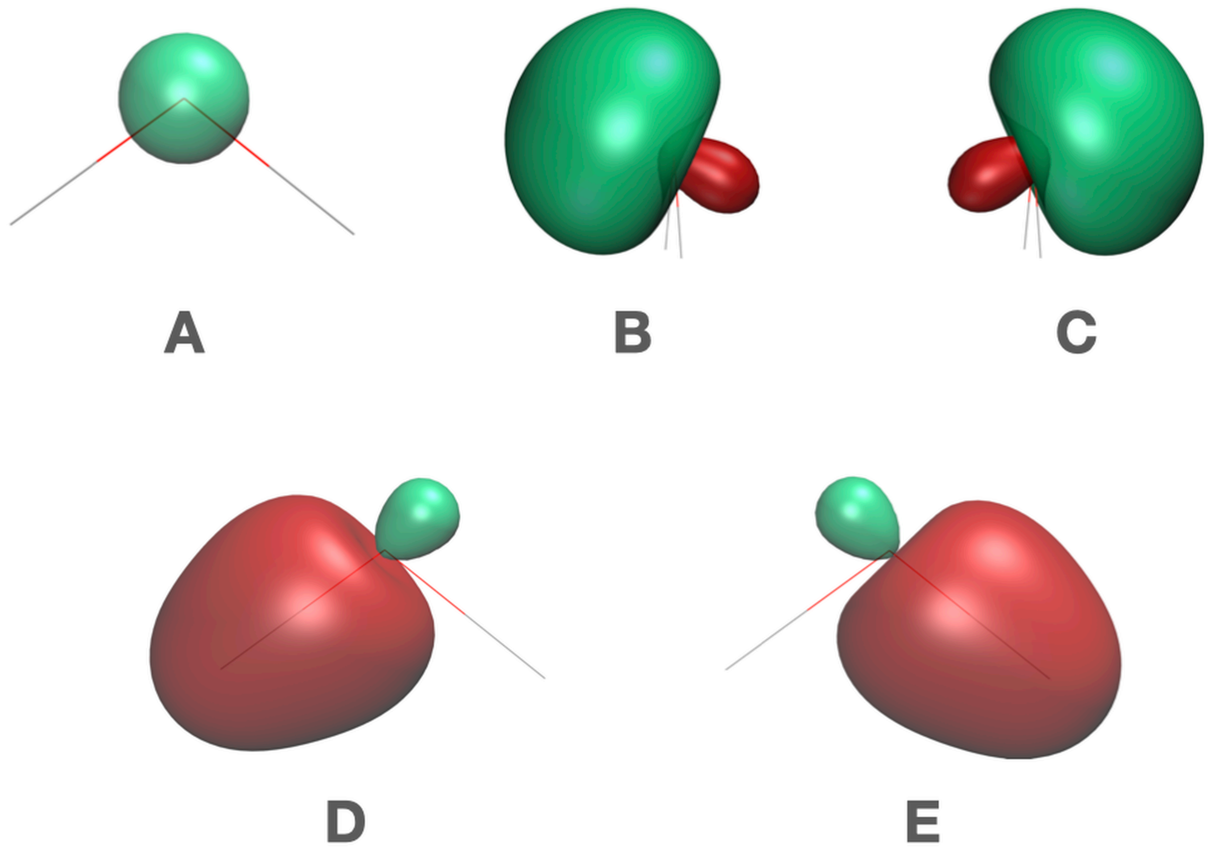

Figure S1. Extremely Localized Molecular Orbitals computed for water using a localization scheme corresponding to the Lewis structure: (A) ELMO associated with the core electrons of the oxygen atom, (B, C) ELMOs corresponding to the two oxygen-lone-pairs, (D, E) ELMOs describing the two O-H bonds of the water molecule. All the orbitals were computed using the cc-pVDZ basis-set and were plotted considering the 0.2 a.u. isosurface.

S1.2 Transferability and rotation of ELMOs.

Because of their strict localization, ELMOs are molecular orbitals that we can easily export from molecule to molecule.[3,4] In particular, we can transfer them from a model system (which is usually a small molecule on which the molecular orbital is originally computed) to the target system that we aim to study. This can be accomplished by exploiting a strategy proposed by Philipp and Friesner.[5]

This technique allows the definition of a matrix **P** that rotates the coefficients of the ELMOs (see equation (S1)) in the geometry of the model molecule to the coefficients of the ELMOs in the geometry of the target system. To get this rotation matrix, we need the definition of i) a reference frame (**a**, **c**, **d**) in the model molecule and of ii) a reference frame (**a'**, **c'**, **d'**) in the target system (see Figure S2).

These two reference frames result from the choice of two atomic triads (one for the model molecule and one for the target system). By indicating the triads for the model and target systems as (A_1, A_2, A_3) and (A_1', A_2', A_3'), respectively, the vectors defining the two reference frames are: **a** (**a'**), which is the position vector of A_2 (A_2') relative to A_1 (A_1') (see Figure S2), **c** (**c'**) and **d** (**d'**), which are given by

$$\begin{cases} \mathbf{c} = \mathbf{a} \times \mathbf{b} & (\mathbf{c}' = \mathbf{a}' \times \mathbf{b}') \\ \mathbf{d} = \mathbf{c} \times \mathbf{a} & (\mathbf{d}' = \mathbf{c}' \times \mathbf{a}') \end{cases} \qquad \text{(S5)},$$

with **b** (**b'**) as the position vector of A_3 (A_3') with respect to A_1 (A_1') (see again Figure S2).

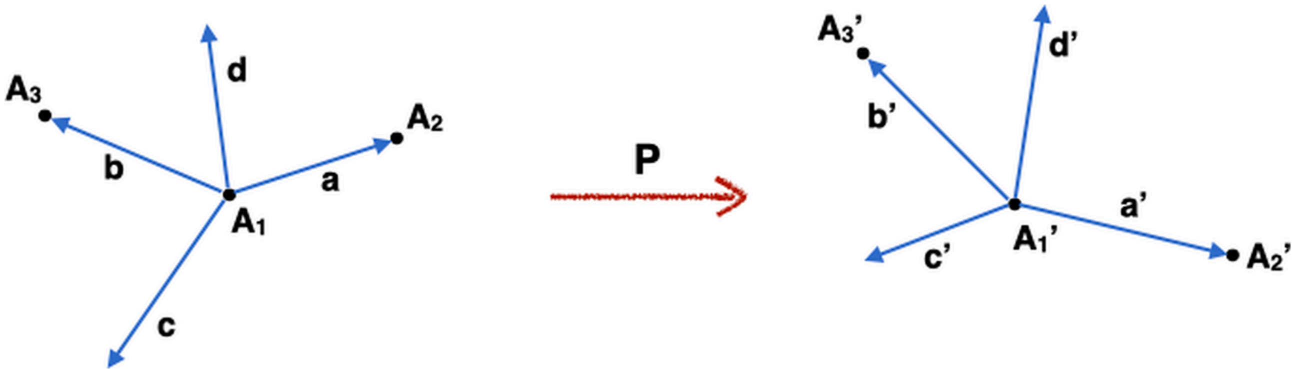

Figure S2. Schematic representation of the reference frames and of the atomic triads that one needs in order to define the rotation matrix **P** for the transformation of the ELMOs from the geometry of the model system (left) to the geometry of the target molecule (right).

For ELMOs localized on a single atom (i.e., ELMOs corresponding to core or lone-pair electrons) the atomic triads are constituted by the atom on which the ELMO is localized and, usually, by two other bonded atoms. For ELMOs localized on two-center bonds, the triads are given by the atoms forming the bond with the addition of an atom describing the local dissymmetry of the bond under exam.[6] For ELMOs localized on three centers (e.g., ELMOs describing situations in which it is important to account for the delocalized nature of the electronic structure, such as in peptide bonds or aromatic rings), the triads of atoms are automatically defined. For ELMOs localized on more than three atoms, it is impossible to define triads (and reference frames) that simultaneously take into account the

orientation of all the atoms in the subunit. This is the reason why, in the current libraries, all ELMOs are localized at the largest on three atomic centers (see next subsection).

The above-mentioned rotation matrix **P** is a matrix that also allows the preparation of all the other matrices that are necessary for the rotation of all kinds of basis functions and associated ELMO coefficients. In fact, neglecting the *s*-type basis functions (which are invariant to rotations because they have spherical symmetry), it is easy to show that *p*-type atomic orbitals (and the related coefficients) can be rotated according to the above-mentioned matrix **P**, while basis functions (and corresponding coefficients) with angular momentum greater than 1 can be transformed using matrices that can be expressed as a function of **P**.[3]

S1.3 Libraries of Extremely Localized Molecular Orbitals

Exploiting the transferability of ELMOs, libraries of Extremely Localized Molecular Orbitals have been recently assembled.[7] The ELMO databanks allow the description of all the possible fragments of water molecule and of the twenty natural amino acids in all their possible protonation states and forms (namely, N-terminal, C-terminal and non-terminal forms). The ELMOs in the databases were determined on suitable model molecules by considering the chemical environment of the fragments. Moreover, the ELMO libraries were assembled for five standard quantum chemistry basis sets (i.e., 6-31G, 6-311G, 6-31G(d,p), 6-311G(d,p) and cc-pVDZ) and include molecular orbitals absolutely localized on one-atom fragments (corresponding to core or lone pair electrons), two-atom subunits (corresponding to bonding electrons), but also molecular orbitals strictly localized on three-atom fragments, which enable to describe the delocalization of the electronic structure in some particular cases (e.g., in carboxylic groups, peptide bonds or aromatic rings).

The software *ELMOdb*[7] allows the transfer of ELMOs from the libraries to the target structures. By analyzing the PDB files of polypeptides or proteins, it carries out the transfer procedure for each subunit of all residues. The *ELMOdb* software also reads tailor-made ELMOs corresponding to fragments of molecules that are not included in the databanks (e.g., ELMOs describing fragments of ligands when we have protein-ligand complexes). These ELMOs are calculated on suitable model molecules and stored in folders from which the *ELMOdb* program can read them.

The ELMO libraries and the associated *ELMOdb* program are available upon motivated request to the one of the corresponding authors of the present paper (A.G.).

S2. Theoretical Details on the preliminary orthogonalization procedure of the QM/ELMO method

As described in the main text, the orthogonalization procedure of the QM/ELMO method entails the following three steps:[8,9]

1. Löwdin orthonormalization of the transferred extremely localized molecular orbitals.
2. Orthogonalization of the QM basis functions against the Löwdin orthonormalized ELMOs.
3. Canonical orthogonalization of the QM basis functions resulting from the previous step.

Since the first step exploits the Löwdin orthogonalization, the original ELMOs only partially delocalize. This leads to a new set of equivalent molecular orbitals with small tails in regions that are very close to the fragments on which the starting ELMOs were strictly localized.

The second step projects out the Löwdin orthonormalized ELMOs from the original basis functions of the QM region. This is equivalent to the following matrix transformation:

$$\tilde{\boldsymbol{\chi}} = \boldsymbol{\chi}\,\mathbf{T} \qquad \text{(S6)},$$

with $\boldsymbol{\chi} = [|\chi_1\rangle, |\chi_2\rangle, \dots, |\chi_M\rangle]$ as the $1 \times M$ array of the original supermolecular basis set (which include all the basis functions of the QM and ELMO regions), $\tilde{\boldsymbol{\chi}} = [|\tilde{\chi}_1\rangle, |\tilde{\chi}_2\rangle, \dots, |\tilde{\chi}_{M_{QM}}\rangle]$ as the $1 \times M_{QM}$ array of the basis functions associated with the only QM subsystem after the orthogonalization, and $\mathbf{T}$ as the $M \times M_{QM}$ transformation matrix with elements that can be expressed as follows

$$T_{\nu\mu} = \left[1 - \sum_{i=1}^{N_{ELMO}} \left(S_{\mu i}\right)^2\right]^{-1/2} \left[\delta_{\nu\mu} - \sum_{i=1}^{N_{ELMO}} C_{\nu i}^{\perp}\, S_{\mu i}\right] \qquad \text{(S7)}.$$

In equation (S7), $S_{\mu i}$ is the overlap integral between the original basis function $|\chi_\mu\rangle$ and the i-th orthonormalized ELMO, $\delta_{\nu\mu}$ the Kronecker delta, and $C_{\nu i}^{\perp}$ the coefficient of the i-th Löwdin orthonormalized ELMO for the starting basis function $|\chi_\nu\rangle$.

Finally, the third step of the orthogonalization procedure is the canonical orthogonalization of the transformed basis functions $\tilde{\boldsymbol{\chi}}$:

$$\boldsymbol{\chi}' = \tilde{\boldsymbol{\chi}}\,\mathbf{W} \qquad \text{(S8)},$$

with $\boldsymbol{\chi}' = [|\chi'_1\rangle, |\chi'_2\rangle, \dots, |\chi'_{M_{QM}}\rangle]$ as the $1 \times M_{QM}$ array of the final orthonormal basis functions for the QM region, and $\mathbf{W}$ as an $M_{QM} \times M_{QM}$ matrix with elements given by

$$W_{ij} = \frac{\tilde{V}_{ij}}{\tilde{v}_j^{\,1/2}} \qquad \text{(S9)},$$

where $\tilde{V}_{ij}$ is the i-th component of the j-th eigenvector, and $\tilde{v}_j$ the j-th eigenvalue of $\tilde{\mathbf{S}}$ (which is the matrix of the overlap integrals between the transformed basis functions $\tilde{\boldsymbol{\chi}}$).

The second and third steps can be combined into one that brings from the original (non-orthogonal) supermolecular basis set $\boldsymbol{\chi}$ for the QM and ELMO regions to the final orthonormal basis functions $\boldsymbol{\chi}'$ for the only QM subsystem:

$$\boldsymbol{\chi}' = \boldsymbol{\chi}\,\mathbf{B} \quad \text{(S10)}$$

Equation (S10) is exactly equation (4) in the main text, where we pointed out the fundamental role of the matrix **B** in the QM/ELMO self-consistent field algorithm. Here, it is also worth noting that the global transformation **B** can be also seen as the result of the following matrix product:

$$\mathbf{B} = \mathbf{T}\,\mathbf{W} \quad \text{(S11)},$$

where **T** and **W** are defined by equations (S7) and (S9), respectively.

S3. Supplementary figures

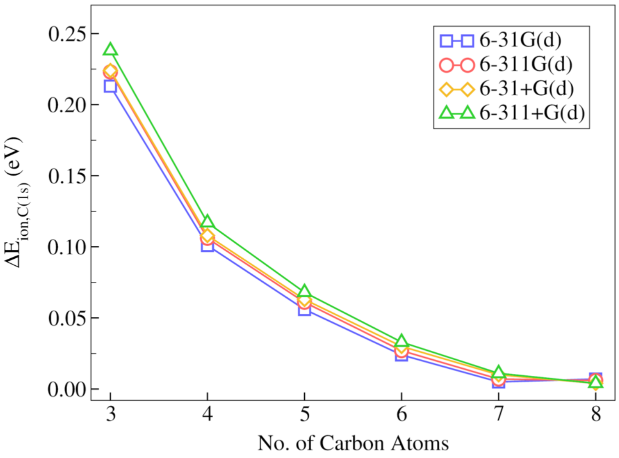

Figure S3. Absolute deviations of the IMOM/ELMO 1s core-ionization energies for the terminal carbon atom of decane from the reference fully IMOM values ($\Delta E_{ion,C(1s)}$), as a function of the number of carbon atoms gradually included in the quantum mechanical region of the QM/ELMO calculations. Only the results of the computations performed with the B3LYP functional are reported.

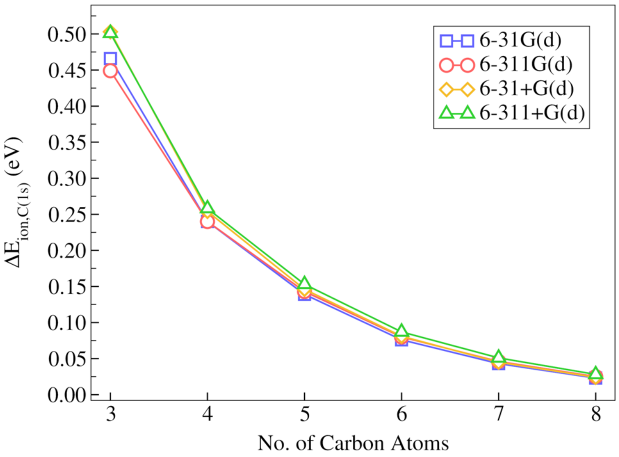

Figure S4. Absolute deviations of the IMOM/ELMO 1s core-ionization energies for the carbonyl carbon atom of 2-decanone from the reference fully IMOM values ($\Delta E_{ion,C(1s)}$), as a function of the number of carbon atoms gradually included in the quantum mechanical region of the QM/ELMO calculations. Only the results of the computations performed with the B3LYP functional are reported.

S4. Supplementary tables

Table S1. Absolute deviations (in eV) of the IMOM/ELMO 1s core-ionization energies for the terminal carbon atom of decane from the reference fully IMOM values ($\Delta E_{ion,C(1s)}$), as a function of the number of carbon atoms progressively included in the quantum mechanical subsystem of the performed QM/ELMO computations. Last row with the core-ionization energies (in eV) obtained at fully IMOM level. Only the results of the calculations with the PBE0 functional are reported.

No. of carbon atoms	$\Delta E_{ion,C(1s)}$ (eV)			
	6-31G(d)	6-311G(d)	6-31+G(d)	6-311+G(d)
3	0.226	0.235	0.237	0.251
4	0.110	0.116	0.116	0.126
5	0.063	0.069	0.070	0.076
6	0.030	0.034	0.036	0.040
7	0.010	0.013	0.014	0.016
8	-0.003	-0.001	0.000	0.001
Fully IMOM	291.363	290.037	291.409	290.031

Table S2. Absolute deviations (in eV) of the IMOM/ELMO 1s core-ionization energies for the terminal carbon atom of decane from the reference fully IMOM values ($\Delta E_{ion,C(1s)}$), as a function of the number of carbon atoms progressively included in the quantum mechanical subsystem of the performed QM/ELMO computations. Last row with the core-ionization energies (in eV) obtained at fully IMOM level. Only the results of the calculations with the B3LYP functional are reported.

No. of carbon atoms	$\Delta E_{ion,C(1s)}$ (eV)			
	6-31G(d)	6-311G(d)	6-31+G(d)	6-311+G(d)
3	0.213	0.223	0.224	0.238
4	0.101	0.106	0.108	0.117
5	0.056	0.061	0.063	0.068
6	0.024	0.027	0.030	0.033
7	0.005	0.007	0.010	0.011
8	-0.007	-0.006	-0.004	-0.004
Fully IMOM	292.057	290.628	292.118	290.622

Table S3. Relative discrepancies (in %) of the IMOM/ELMO 1s core-ionization energies for the terminal carbon atom of decane with respect to the reference fully IMOM values, as a function of the number of carbon atoms progressively included in the quantum mechanical subsystem of the performed QM/ELMO computations. Only the results of the calculations with the B3LYP functional are reported.

No. of carbon atoms	IMOM/ELMO relative discrepancies for core-ionization energies (%)			
	6-31G(d)	6-311G(d)	6-31+G(d)	6-311+G(d)
3	0.073	0.077	0.077	0.082
4	0.034	0.037	0.037	0.040
5	0.019	0.021	0.022	0.024
6	0.008	0.009	0.010	0.011
7	0.002	0.002	0.003	0.004
8	-0.003	-0.002	-0.001	-0.001

Table S4. Absolute deviations (in eV) of the IMOM/ELMO 1s core-ionization energies for the carbonyl carbon atom of 2-decanone from the reference fully IMOM values ($\Delta E_{ion,C(1s)}$), as a function of the number of carbon atoms progressively included in the quantum mechanical subsystem of the performed QM/ELMO computations. Last row with the core-ionization energies (in eV) obtained at fully IMOM level. Only the results of the calculations with the PBE0 functional are reported.

No. of carbon atoms	$\Delta E_{ion,C(1s)}$ (eV)			
	6-31G(d)	6-311G(d)	6-31+G(d)	6-311+G(d)
3	0.473	0.458	0.512	0.510
4	0.251	0.251	0.267	0.269
5	0.147	0.150	0.155	0.160
6	0.083	0.086	0.088	0.093
7	0.048	0.051	0.051	0.055
8	0.027	0.029	0.029	0.032
Fully IMOM	294.304	292.914	294.415	292.964

Table S5. Absolute deviations (in eV) of the IMOM/ELMO 1s core-ionization energies for the carbonyl carbon atom of 2-decanone from the reference fully IMOM values ($\Delta E_{ion,C(1s)}$), as a function of the number of carbon atoms progressively included in the quantum mechanical subsystem of the performed QM/ELMO computations. Last row with the core-ionization energies (in eV) obtained at fully IMOM level. Only the results of the calculations with the B3LYP functional are reported.

No. of carbon atoms	$\Delta E_{ion,C(1s)}$ (eV)			
	6-31G(d)	6-311G(d)	6-31+G(d)	6-311+G(d)
3	0.466	0.449	0.503	0.501
4	0.240	0.240	0.254	0.258
5	0.139	0.143	0.146	0.153
6	0.076	0.080	0.081	0.087
7	0.043	0.046	0.045	0.051
8	0.023	0.025	0.024	0.028
Fully IMOM	294.919	293.462	295.063	293.522

Table S6. Relative discrepancies (in %) of the IMOM/ELMO 1s core-ionization energies for the carbonyl carbon atom of 2-decanone with respect to the reference fully IMOM values, as a function of the number of carbon atoms progressively included in the quantum mechanical subsystem of the performed QM/ELMO computations. Only the results of the calculations with the B3LYP functional are reported.

No. of carbon atoms	IMOM/ELMO relative discrepancies for core-ionization energies (%)			
	6-31G(d)	6-311G(d)	6-31+G(d)	6-311+G(d)
3	0.158	0.153	0.170	0.171
4	0.081	0.082	0.086	0.088
5	0.047	0.049	0.050	0.052
6	0.026	0.027	0.027	0.030
7	0.015	0.016	0.015	0.017
8	0.008	0.009	0.008	0.010

S5. References for the Supporting Information

[1] Stoll, H.; Wagenblast, G.; Preuss, H. On the Use of Local Basis Sets for Localized Molecular Orbitals. *Theor. Chim. Acta* **1980**, *57*, 169−178.

[2] Sironi, M.; Genoni, A.; Civera, M.; Pieraccini, S.; Ghitti, M. Extremely Localized Molecular Orbitals: Theory and Applications. *Theor. Chem. Acc.* **2007**, *117*, 685-698.

[3] Meyer, B.; Guillot, B.; Ruiz-Lopez, M. F.; Genoni, A. Libraries of Extremely Localized Molecular Orbitals. 1. Model Molecules Approximation and Molecular Orbitals Transferability. *J. Chem. Theory. Comput.* **2016**, *12*, 1052-1067.

[4] Meyer, B.; Guillot, B.; Ruiz-Lopez, M. F.; Jelsch, C.; Genoni, A. Libraries of Extremely Localized Molecular Orbitals. 2. Comparison with the Pseudoatoms Transferability. *J. Chem. Theory. Comput.* **2016**, *12*, 1068-1081.

[5] Philipp, D. M.; Friesner, R. A. Mixed Ab Initio QM/MM Modeling Using Frozen Orbitals and Tests with Alanine Dipeptide and Tetrapeptide. *J. Comput. Chem.* **1999**, *20*, 1468-1494.

[6] Ferré, N.; Assfeld, A.; Rivail, J.-L. Specific Force Field Parameters Determination for the Hybrid Ab Initio QM/MM LSCF Method. *J. Comput. Chem.* **2002**, *23*, 610-624.

[7] Meyer, B.; Genoni, A. Libraries of Extremely Localized Molecular Orbitals. 3. Construction and Preliminary Assessment of the New Databanks. *J. Phys. Chem. A* **2018**, *122*, 8965-8981.

[8] Macetti, G.; Genoni, A. Quantum Mechanics/Extremely Localized Molecular Orbital Method: A Fully Quantum Mechanical Embedding Approach for Macromolecules. *J. Phys. Chem. A* **2019**, *123*, 9420-9428.

[9] Macetti, G.; Wieduwilt, E. K.; Assfeld, X.; Genoni. A. Localized Molecular Orbital-Based Embedding Scheme for Correlated Methods. *J. Chem. Theory Comput.* **2020**, *16*, 3578-3596.
